# Pneumatic–Cable-Hybrid-Driven Multi-Mechanism End Effector and Cross-Surface Validation

**DOI:** 10.3390/biomimetics11020140

**Published:** 2026-02-12

**Authors:** Zhongyuan Wang, Zhiyuan Weng, Peiqing Zhang, Wei Jiang, Nan Deng, Zhouyi Wang

**Affiliations:** 1 Research and Development Center, China Unicom Internet of Things Co., Ltd., Nanjing 210006, China; jiangw32@chinaunicom.cn (W.J.);; 2 College of Mechanical and Electrical Engineering, Nanjing University of Aeronautics and Astronautics, Nanjing 210016, China; 3 Embodied AI Innovation Center, China Unicom Digital Technology Co., Ltd., Beijing 100032, China; 4 Shenzhen Research Institute, Nanjing University of Aeronautics and Astronautics, Shenzhen 518063, China

**Keywords:** multi-mechanism coupled adhesion, claw, gecko-like robot, flexible end effector, rough curved surface

## Abstract

Wall-climbing robots are increasingly required for applications in aerospace, high-altitude operations, and complex environmental monitoring, where they must maintain reliable adhesion and continuous mobility across surfaces with rapidly changing material properties and roughness. Achieving these demands requires lightweight systems with end effectors that integrate multi-surface adaptability and load-carrying capacity. Current single adhesion mechanisms are typically effective only under specific wall conditions, making it challenging to achieve stable, continuous adhesion and detachment on surfaces with significantly different roughness. To address this limitation, we propose a flexible, multi-mechanism coupled end effector driven by a pneumatic–cable hybrid system, integrating two complementary adhesion mechanisms—claw-based interlocking and vacuum suction—into a unified flexible structure. First, we develop the overall structural framework of the end effector and conduct finite element simulations to analyze key structural parameters of the telescopic cavity. We then establish a contact force model between the claw and vertical rough surfaces to clarify the interlocking adhesion mechanism and determine critical geometric parameters. Based on these analyses, a cable-driven adjustment mechanism is introduced to enable dynamic self-adaptation and assist with load-bearing during adhesion, enhancing the stability and load-carrying capacity under varying wall conditions. On rough surfaces, the end effector achieves reliable adhesion through claw interlocking, while on smooth surfaces, it maintains stable attachment through vacuum suction. Furthermore, it supports seamless switching between adhesion modes on different surfaces. When integrated into a wall-climbing robot, the system enables stable adhesion and detachment on both rough and smooth surfaces, providing a feasible solution for the lightweight, integrated design of end effectors for multi-surface adaptive wall-climbing robots.

## 1. Introduction

With the advancement of urbanization and industrial restructuring, the demand for wall-climbing robots in applications such as aerospace exploration, high-altitude operation maintenance, nuclear industry inspection, and field reconnaissance is increasing. The end effector is a key component in robot–environment interactions, as it generates the adhesion forces necessary to support the robot’s movement on walls [1,2]. According to different adhesion mechanisms, common end adhesion methods include vacuum suction [3,4], magnetic adhesion [5], claw/micro-spine adhesion [6,7], and bio-inspired dry adhesion [8,9], etc. Among these, magnetic adhesion has a simple structure but is limited to magnetically conductive materials; dry adhesion performs well on specific smooth walls, but its cost, durability, and self-cleaning ability still need improvement. In the field of vacuum suction, research focuses mainly on the efficiency improvement of suction structures and their adaptability to complex surfaces. Bamotra et al. (2019) proposed a design using a porous vacuum structure instead of multi-suction cups, achieving a maximum suction force of 40 N [10]; Koivikko et al. (2021) developed a switchable magnetic soft suction cup gripper, which realizes soft–rigid state switching via magnetorheological fluid to improve the grasping ability for curved, rough, wet objects and thin films [11]; Wang et al. revealed the mechanism of fish head sucker adhesion to complex surfaces during long-term attachment and developed a corresponding end effector [12]; Hwang et al. proposed an octopus-inspired sucker micro-structure combined with a pneumatic mesh bending actuator, realizing switchable grasping and separation in dry, wet, and underwater environments [13]; Wang et al. combined vacuum negative pressure with dry adhesion and developed a flexible adhesive gripper with active detachment based on a fast-response pneumatic bending actuator [14]. Vacuum suction has high engineering maturity and controllability on smooth walls such as glass and acrylic, but it is sensitive to the sealing integrity—leakage easily occurs on surfaces with rough particles, pores, or cracks, leading to reduced reliability. Moreover, a system-level modular architecture and sensor-driven real-time coordination have been quantitatively validated as critical enablers for reliable performance in complex multi-actuator robotic platforms (e.g., electro-hydraulic humanoids) [15].

When the operational environment transitions from low-roughness walls to rough, uneven, and irregular surfaces, relying solely on negative pressure, suction often fails to provide reliable adhesion. As a result, interlocking adhesion mechanisms must be introduced to supplement suction. For rough, uneven, and irregular surfaces, claw-based interlocking adhesion offers advantages in load-carrying capacity and energy efficiency due to its interlocking mechanism. Saunders et al. developed an adhesion mechanism inspired by the climbing behavior of cockroach claws, enabling a hexapod robot to ascend a 55° inclined hardwood board and climb a vertical tree trunk at 1 cm/s. Subsequent improvements allowed the robot to adapt to brick surfaces, tree trunks (cylindrical rough surfaces), and climb wooden utility poles at speeds of up to 21 cm/s [16,17,18]. Parness et al. (2011) applied micro-spine grippers to Mars exploration concepts, developing the LEMUR IIB robot—its end is composed of hundreds of micro-spine toes, which can share loads and improve adhesion probability; tests showed that about 10–30% of the toes can interlock with rock surfaces and generate a tensile force of approximately 107 N, and optimization enabled adaptation to irregular geometric boulder surfaces [19,20,21]. Later, Nagaoka et al. proposed a passive micro-spine gripper for rough rock adhesion [22]; Backus Spencer B et al. developed the JPL-Nautilus underactuated micro-spine gripper for underwater rock sampling, which can generate a grasping force of approximately 450 N [23]; Ji et al. developed a hexapod claw-based wall-climbing robot, which realizes claw opening and closing via cable tension and achieves climbing on grid surfaces and 60° slopes [24]. In addition, ReachBot (2022–2024) uses tendon-driven micro-spine grippers for planetary cave exploration, with each finger providing approximately 7.5 N of grasping force [25,26]; the Ding team from Beihang University of Aeronautics and Astronautics proposed a multi-modal rock-climbing gripper with both adhesion and support functions, which can generate approximately 43 N of normal adhesion force and 49 N of tangential adhesion force on basalt surfaces, and this enables MARCRot to climb 90°vertical rock walls at 2.5 mm/s [27]. Although claw-based interlocking adhesion has advantages such as high load-carrying capacity, low energy consumption, and low noise on rough walls, its effective adhesion depends on the presence of interlockable features and adhesion points, making it difficult to achieve stable adhesion on smooth walls.

The existing research indicates that a single adhesion mechanism is typically advantageous only under specific surface conditions: negative pressure suction is vulnerable to seal failure, while interlocking adhesion is limited by the density and geometric distribution of interlockable features. As a result, it is challenging to meet the operational demands of frequent ‘smooth–rough’ surface transitions in real-world scenarios. In this context, multi-modal adhesion has emerged as a key approach to expand the effective working range. For example, Wang et al. proposed a bio-inspired attachment system that integrates van der Waals adhesion with negative pressure suction [28]. Liu et al. integrated spine wheels, adhesive belts, and eddy suction cups into a unified platform [15], enhancing the overall adaptability through the coordination or switching of multiple attachment subsystems. However, most existing multi-modal solutions rely on the mechanical stacking of multiple attachment mechanisms to achieve surface coverage, which inevitably increases the weight, volume, and control complexity. Additionally, during the transition from rough to smooth surfaces, these solutions often face challenges with attachment continuity and reduced safety margins. A key challenge remains in balancing high load-carrying capacity and strong adaptability within a single end effector structure, while ensuring stable adhesion during cross-surface transitions.

Given the comprehensive requirements for end effector performance, lightweight design, load-carrying capacity, and environmental adaptability, as well as the inherent trade-off between load-carrying capacity and adaptability, this paper proposes and develops a flexible bio-inspired end effector driven by a pneumatic–cable hybrid system, which integrates two complementary adhesion mechanisms (claw-based interlocking adhesion and vacuum suction) into a unified end structure. Focusing on this end effector, this paper systematically conducts structural design and mechanical modeling analysis and verifies its stable adhesion and detachment performance on both smooth and rough walls via wall-climbing robot experiments. The experimental results show that this end effector can achieve reliable adhesion on both ultra-smooth walls (Ra < 0.1 μm) and highly rough walls (Ra > 1 μm), enabling a wall-climbing robot to complete continuous cross-surface climbing under low-pressure drive conditions. This research provides a new idea for the integrated and lightweight design of end effectors for multi-surface adaptive wall-climbing robots.

## 2. Design of a Multi-Mechanism Coupled End Effector for Cross-Roughness Substrates

**Figure 1:**
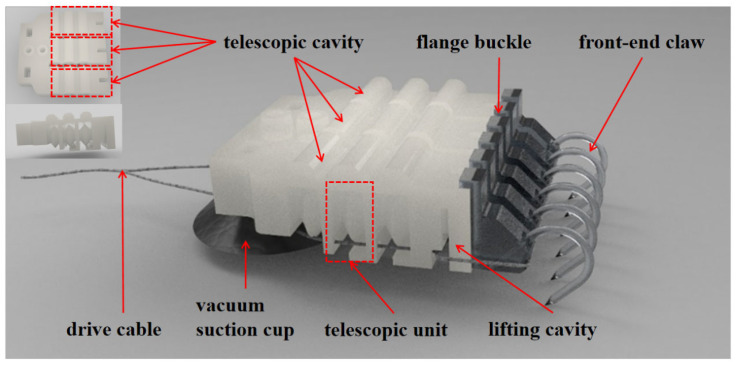
Schematic diagram of the flexible bionic attachment device.

The multi-mechanism coupled end effector developed in this paper is suitable for cross-scale rough surfaces (i.e., it has certain adaptability to both ultra-smooth walls with Ra < 0.1 μm and highly rough walls with Ra > 1 μm). It adopts a pneumatic–cable hybrid drive mode, and its overall structure mainly consists of three parallel flexible corrugated telescopic cavities, a root vacuum suction cup, end claws, and a cable assembly for posture and load adjustment (as shown in the Figure 1). Each flexible telescopic cavity is composed of three corrugated telescopic units connected in series with a front upward-lifting cavity, which has strong active deformation capability and can significantly improve the end effector’s adaptability to wall geometric shapes. On rough walls, adhesion is mainly achieved via interlocking and sliding friction between the claws at the end of the telescopic cavity and the raised particles on the surface. Each telescopic cavity is equipped with two independent claws at the end, which helps adapt to irregular rough surfaces and achieve uniform load distribution. When driven by positive pressure to expand, the front upward-lifting cavity produces a lifting deformation, which is used to realize active detachment of the claws from the rough substrate. The interior of this upward-lifting cavity is divided into two independent air channels, corresponding to the two claws respectively, forming a dual-branch structure similar to the tarsal segment of beetles [29], which reduces the probability that claws are difficult to detach after embedding into rough particles during positive-pressure detachment. On smooth walls, adhesion is achieved via the vacuum suction cup located at the central position of the end effector’s bottom. Considering structural compactness and the layout of bio-inspired telescopic cavities, the suction cup adopts an elliptical external design. Its bottom height is between the tip of the lowermost claw and the flexible flap at the bottom of the telescopic cavity, which ensures suction performance while avoiding structural interference between the smooth-surface and rough-surface adhesion modes.

### 2.1. Structural Parameters of the End Claw

In nature, climbing organisms mostly face rough, unstructured and irregular walls. To better adapt to survival, these organisms have evolved flexible adhesion ends with excellent adaptability. Professor Dai established a spherical contact model between the tip of insect claws and horizontal rough surfaces [30], which simplifies the contact analysis between insect claw tips and sandpaper surface particles into a contact problem between two spherical surfaces. Similar simplified claw–substrate contact representations have been widely used to provide mechanistic, qualitative explanations of attachment enhancement on rough surfaces (e.g., claw–pad synergy) [31]. However, the working condition addressed in this paper mainly involves adhesion to vertical walls, so the effects of tangential and normal forces during adhesion need to be considered. Under gravity, the tangential force at the claw tip mainly undertakes the load-carrying function, which is used to balance the robot’s own weight and additional loads. When moving vertically upward, an overturning moment will be generated, leading to the robot body tipping over. Thus, the effect of the normal force generated by the claw tip contact needs to be considered.

**Figure 2:**
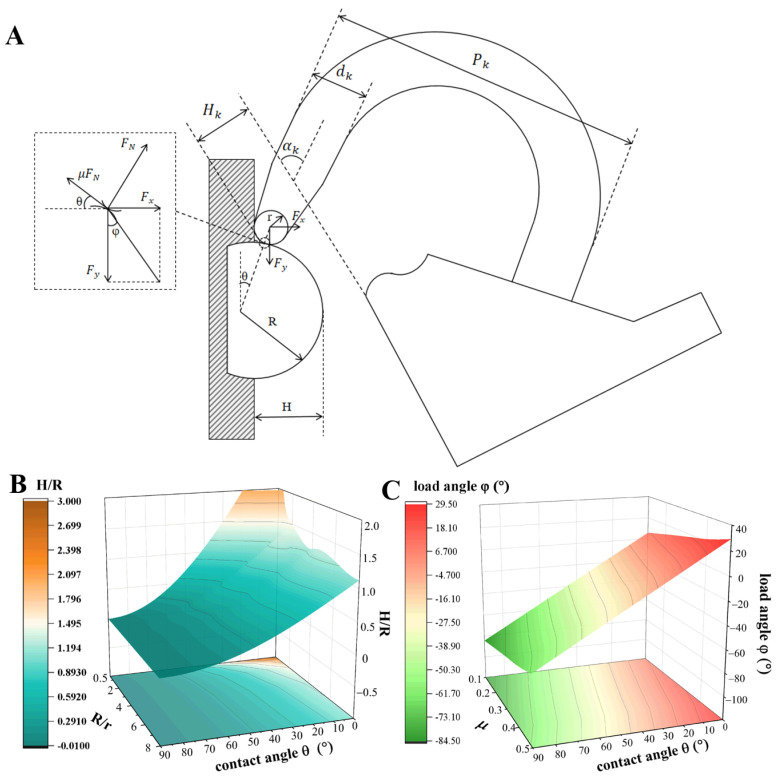
(**A**): Definition of claw parameters and spherical contact model between claw tip and vertical rough surface; (**B**,**C**): variation curves of load angle *φ* with claw tip radius *r*, protrusion radius *R*, and friction coefficient *μ*.

As shown in Figure 2A, the contact between the claw tip and the vertical rough wall is also regarded as the contact between two spherical surfaces. Define the following parameters: in the initial position, the angle between the claw axis and the bottom of the flange buckle is $\alpha_{k}$; the distance from the bottom of the claw tip to the bottom of the flange buckle is $H_{k}$; the claw diameter is $d_{k}$; the claw width is $P_{k}$; the radius of the claw tip is *r*; the radius of the raised particle on the rough surface is *R*; the contact angle between the claw and the rough surface is θ; the load angle is φ; the height of the raised particle on the rough surface is *H*; the overturning force on the claw tip during adhesion is $F_{x}$; the tangential component of gravity on the claw tip during adhesion is $F_{y}$; the normal support force from the raised particle on the claw tip is $F_{N}$; and the friction coefficient between the claw tip and the surface of raised particle is μ.

The contact angle θ can be obtained from the claw tip radius *r*, the raised particle radius *R*, and the raised particle height *H*:

(1) $$\sin\theta =\frac{r+R-H}{r+R}=1-\frac{H}{r+R}$$

It can be obtained from force balance that

(2) $$F_{x}+F_{N}\sin\theta =\mu F_{N}\cos\theta$$

(3) $$F_{y}=\mu F_{N}\sin\theta +F_{N}\cos\theta$$

Combining the above two equations,

(4) $$\frac{F_{x}}{F_{y}}=\frac{\mu \cos\theta -\sin\theta }{\mu \sin\theta +\cos\theta }=\frac{\mu -\tan\theta }{\mu \tan\theta +1}$$

(5) $$\tan\varphi =\frac{F_{x}}{F_{y}}$$

From Equation (4), when θ>arctanμ (i.e., the claw contact angle θ is large), the claw tip cannot achieve interlocking adhesion. Combining the above two equations,

(6) φ=arctanμ−θ

From Equations (4) and (6), when the bonding strength between the claw tip and the particles on the adhesion surface is sufficient (i.e., the claw tip does not fail and the particles on the adhesion surface do not fall off), the maximum load angle *φ* of the claw tip is related to the contact angle *θ* and the contact friction coefficient *μ* between the claw and the wall. The effects of claw tip radius *r*, raised particle radius *R*, and contact friction coefficient *μ* on the load angle *φ* are shown in Figure 2B,C. It can be seen that when the raised particle radius is smaller than the claw tip radius (i.e., *R*/*r* ≤ 1), the claw tip is larger than the particle size of the surface to be adhered—at this point, the load angle *φ* < 0, meaning that even if the friction coefficient is large, the claw cannot generate normal adhesion force on the surface of the raised particle.

When the claw tip radius *r* decreases, the contact angle *θ* decreases and the load angle *φ* increases accordingly; the larger the adhesion force generated by the claw in the normal direction, the more impossible it is to achieve effective adhesion. That is, when the flexible end effector contacts particle protrusions at a certain angle, the claw tip diameter cannot match all gap widths. There will always be smaller protrusions or gaps that prevent the claw from adhering. Therefore, on the premise of ensuring the claw material strength, the smallest possible claw tip diameter should be used. The main parameters of the claw are determined as shown in Table 1.

### 2.2. Structural Design of the Flexible Corrugated Telescopic Cavity

**Figure 3:**
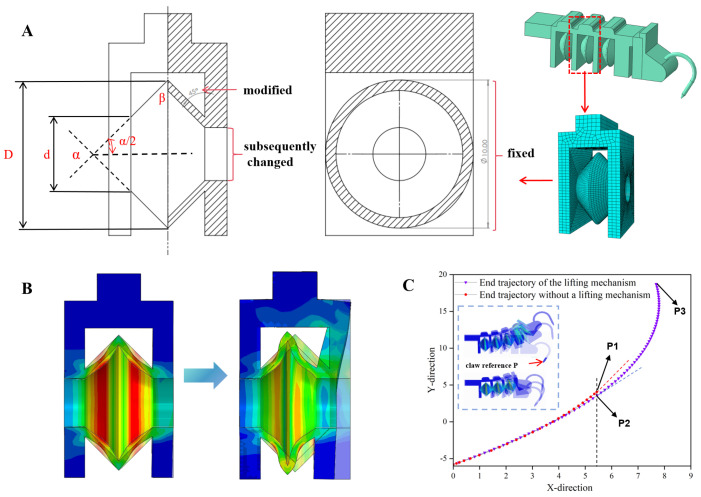
(**A**): Schematic diagram of mesh division for telescopic unit and identification of aspect ratio in telescopic chamber; D: long outer diameter of telescopic chamber; d: short outer diameter of telescopic chamber; α: Total apex angle of the internal cone; β: inclination angle of the modified upper surface) (**B**): simulated detachment state of telescopic unit under the condition of 50 kPa positive pressure; (**C**): schematic diagram of simulated detachment trajectory of claw tip in cases with and without lifting mechanism.

A single corrugated telescopic cavity is composed of three telescopic units connected in series with an end upward-lifting chamber (Figure 3A). There are strain constraint layers and sheaths between the series-connected telescopic units to guide the deformation state of the telescopic units, while enhancing the load-bearing stiffness of the telescopic cavity, appropriately reducing fatigue failure of the structure under cyclic stress and extending its service life.

The deformation state of the telescopic unit can be regarded as follows: the left end of a single telescopic cavity is fixed by the strain constraint layer, the right end is free to deform, and an external pneumatic source applies air pressure to generate a normal tensile force on the right end. Therefore, to understand the effects of various factors on the mechanical performance and deformation state of the telescopic unit structure, the ABAQUS 6.14 (Dassault Systèmes, Providence, RI, USA) was adopted to conduct a finite element simulation analysis of the telescopic unit. Key factors such as the shape, wall thickness, length–diameter ratio, and taper angle of the telescopic cavity were selected for investigation. The effects of the structural parameters of the single telescopic cavity on the deformation state, adhesion force, and other properties under different pneumatic pressure drives were discussed, and parameter optimization was continuously performed to improve the performance of the single cavity. The telescopic cavity structure is mainly responsible for deformation and load-αβ bearing functions.

Simulation results show that under negative pressure, regardless of whether the middle symmetry plane is fixed in an annular shape or the left and right end faces have air inlet/outlet hole shapes and sizes, when the cone angle *α* changes (i.e., the included angle *β* changes), any significant change in the surface area connecting the telescopic cavity to the outside atmosphere will greatly alter the pressure of the outside atmosphere on the telescopic cavity, i.e., greatly change the normal tensile force on the right end of the telescopic cavity. Therefore, within the given structural size range, after determining the size of the air inlet/outlet holes at the left and right ends, increasing the included angle *β* as much as possible can effectively increase the normal tensile force of the telescopic cavity. Thus, the parameter included angle *β* in this section is selected as 36°, i.e., the cone angle *α* is 108°. To obtain a telescopic cavity with ideal deformation and generate higher normal tensile force under negative pressure, the parameters of a single telescopic cavity are determined as shown in Table 2 (Detailed simulation results are in the Supplementary Materials).

Adding an upward-lifting chamber between the end of the telescopic cavity and the claw flange buckle will greatly improve the detachment efficiency of the claw. In the simulation analysis, taking the end of the claw as the reference point, when 50 kPa positive pressure is inputted into the flexible corrugated telescopic cavity, the expansion and deformation effect of the telescopic cavity with the upward-lifting chamber is more obvious than that without the upward-lifting chamber (Figure 3C). As the internal positive pressure gradually stabilizes, the telescopic cavity without the upward-lifting chamber reaches the maximum deformation position at P1, while the telescopic cavity with the upward-lifting chamber reaches the maximum deformation position at P3. The tangent slope at trajectory point A of the cavity without the upward-lifting chamber is greater than that at P2, reflecting that the flexible telescopic cavity with the upward-lifting chamber has a larger tip expansion degree during detachment than that without the upward-lifting chamber, which greatly reduces the failure rate of claw detachment from the gaps between raised particles on rough surfaces. In addition, the contact angle between the claw and the adhesion surface is not less than 45°and not more than 65°; thus, the sum of the initial angle δ of the claw tip and the bending angle γ of the telescopic cavity under working air pressure satisfies the following: 45°≤δ+γ≤65°. In the expected working air pressure range of −40 kPa to −50 kPa, the bending angle γ of the telescopic cavity is [40°, 45°]. Therefore, the initial position of the claw fixed by the claw flange buckle is set such that the claw axis forms a 20° angle with the normal direction, i.e., a 70° angle $\alpha_{k}$ with the horizontal plane (Table 2).

### 2.3. Tendon-Inspired Cable-Driven Layout

**Figure 4:**
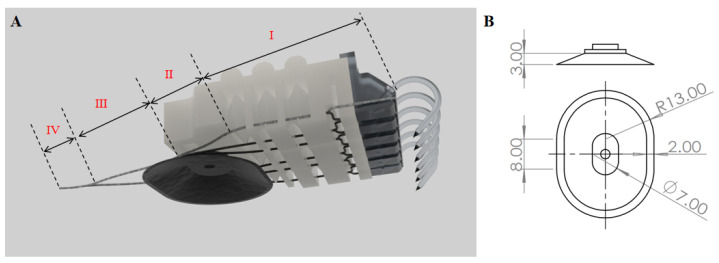
(**A**): Schematic wiring diagram of cable-driven system; (**B**): structural schematic diagram of suction cup.

The climbing behavior of animals on rough walls relies on the synergistic effect of tendons/muscles and claws in the adhesion structure [32]. To further improve the load-carrying capacity of the coupled end effector, a cable-driven structure is integrated into the end effector to simulate tendons, realizing dynamic adaptation and auxiliary grasping functions, and enabling active fitting of the end claw to the climbing wall [33]. The cable-driven system in this paper adopts Dyneema fiber fishing line for its implementation (fishing line specification: 2.5, diameter: 0.261 mm), which has an excellent strength-to-weight ratio. Frictional losses during transmission are minimized as much as possible by reducing the complexity of the transmission path, avoiding large angles of deflection in the path and adopting Teflon polytetrafluoroethylene guide tubes (Figure 4A).

The cable-driven structure is divided into four segments: Segment I consists of three cables passing through the guide holes of the flexible end effector; Segment II involves the three cables being led out and converging on the side of the suction cup; Segment III is the main convergence segment of the six cables on the end effector; Segment IV is the main convergence segment adjusted according to the working condition, for the purpose of adjusting the cable length in this segment sets the reserved length of the cable-driven system. The adjustment aims to make the end effector contract to cover or fit the rough surface as much as possible via the cables under negative pressure, while avoiding interference with suction cup adsorption under positive pressure. The adhesion state on the rough surface under negative pressure serves as the reference point for the reserved cable length: negative values correspond to cable contraction, and positive values correspond to cable relaxation. The reserved length of the cable-driven structure is adjusted appropriately based on actual tests: on rough surfaces, the cables are in a tensioned state to generate clamping force, enabling dynamic adjustment and auxiliary control. On smooth surfaces, during the positive pressure process of the air path where the telescopic cavity and claws are located, the cable-driven system is inactive, and the reserved cable length is long enough to avoid clamping effects that would interfere with smooth-surface adsorption.

Moreover, for rough curved surfaces, the optimal reserved length varies with the surface curvature to ensure adequate conformity and stable load sharing. The experimentally determined reserved length settings for different attachment surfaces are summarized in Table 3 and used consistently in the subsequent adhesion tests.

### 2.4. Parameter Selection of the Flexible Suction Cup

Suction cups are categorized into active and passive types. Passive suction cups require no external energy but have a smaller adsorption force and poor controllability. Active suction cups, when paired with components such as air pumps and solenoid valves, enable precise control and an adjustable adsorption force. Thus, this paper adopts active suction cups. Under negative pressure, the normal force generated by the suction cup is related to the pressure difference between the inner and outer surfaces, the vacuum area, and the internal elastic force of the suction cup; the tangential force is the contact force when relative sliding tends to occur between the suction cup and the surface, influenced by the friction coefficient and the normal force between the suction cup and the contact surface. By establishing a suction-cup-adhesion operation model, conducting actual tests (see Section S2 in Supplementary Materials) and considering the overall coordination and structural compactness of the end effector, the cross-sectional shape of the suction cup is determined to be elliptical, with specific parameters shown in Figure 4B.

## 3. Experimental Methodology

### 3.1. Fabrication and Integration of the Flexible End Effector

**Figure 5:**
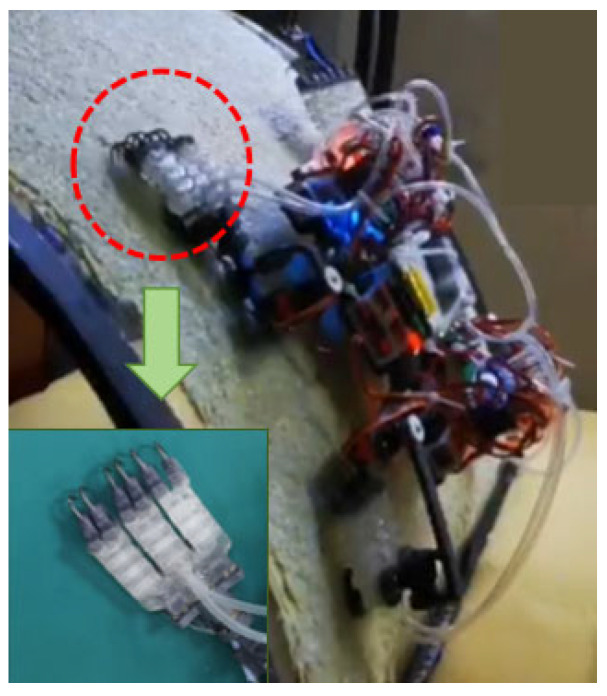
Installation position diagram of attachment device on robot.

The multi-mechanism integrated flexible end effector mainly consists of a flexible telescopic cavity body, a root vacuum suction cup, end claws, and cables for drive and load-bearing. The flexible telescopic cavity was fabricated using a Form 3 stereolithography 3D printer (Formlabs Inc., Somerville, MA, USA). The printing parameters were configured using PreForm software (Formlabs Inc., Somerville, MA, USA). Elastic 50A Resin (Formlabs Inc., Somerville, MA, USA), a flexible photopolymer material with good toughness and elasticity, was selected as the printing material. After printing, the specimens were cleaned and post-cured using a Form Cure unit (Formlabs Inc., Somerville, MA, USA). The Formlabs 3D printing interface PreForm is used to generate supports and set printing parameters, followed by photopolymerization molding. After molding, the tray and printed part are removed and placed in an automatic cleaning machine for cleaning. This process continues until the internal air path of the telescopic cavity is completely clear, preventing residual resin from blocking the channels and affecting subsequent use. The cleaned printed part is then placed in a secondary curing machine for curing. The combined action of light and heat promotes cross-linking reactions between polymer chains in the material, improving its strength, wear resistance, and heat resistance. The suction cup is fabricated using Silicon 50A material, which has a friction coefficient of approximately 0.838 with smooth acrylic surfaces (see Attachment). The end claws are cut from fishhooks to the preset shape and installed in the reserved holes of the flange buckle; glue is used to fix the claws in the preset grooves and at the bottom. For the connection between the suction cup and the flexible end effector, 502 strong adhesive is used. The cable-driven system is matched with the flexible end effector and the single leg of the subsequent robot: the fishing line is cut to an appropriate length and passed through the reserved hole at the lower end of the telescopic cavity; glue is used to fix the middle part of the cable in a U-shaped groove, preventing the cable-driven system from being pulled off during operation. The end effector is equipped with three flexible telescopic cavities (six cables in total); finally, the six cables are converged into a single strand and passed through a Teflon hard tube. A steel cable tensioner is used to release and tighten the cable-driven system, ensuring good contact between the claws and the surface as much as possible (Figure 5).

### 3.2. Integration of the Robot Equipped with the Flexible End Effector

While climbing walls with different roughness levels, the wall-climbing robot must meet the design requirements of miniaturization, lightweight and compactness. The robot adopts a four-leg design, and the entire machine is assembled with the multi-mechanism, integrated flexible end effector (as the adhesion terminal) developed in this paper. After integration, the robot body dimensions are 164.0 mm × 84.0 mm × 39.2 mm, and its weight is 657 g. It operates on a pneumatic–electric hybrid principle: solenoid valves controlled by a PCB board and a micro-diaphragm pump work synergistically to adjust the working state of the end effector during positive/negative pressure switching, meeting different drive requirements. Cable mechanisms driven by motors often have overly complex paths and low transmission efficiency. In this paper, the driving path is limited to the single leg of the robot, and the swing motion of the elbow joint motor itself is used to control the cable-driven system, thereby reducing the demand for driving units. A modular design is adopted: the cable is first threaded through the Teflon rigid tube and then fixed and installed on the single leg of the robot via a snap-fit connection, which ensures the reliability of system integration.

### 3.3. Adhesion Performance Test Platform for the Multi-Mechanism, Integrated Flexible End Effector

**Figure 6:**
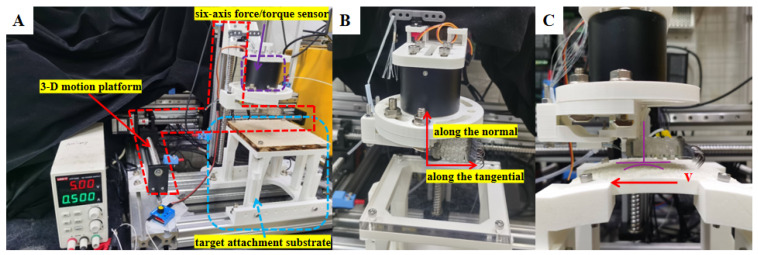
Test platform for adhesion performance of multi-mechanism integrated flexible attachment device. (**A**): Rough flat surface test area; (**B**): smooth flat surface test area; (**C**): rough curved surface test area.

To test the adhesion performance of the multi-mechanism, integrated flexible end effector on surfaces with different roughness, curved surfaces, etc., an experimental platform was built (Figure 6). This platform mainly consists of a 3D mobile platform, a si*x*-axis force sensor, a pneumatic control system, an image-acquisition system, and target adhesion substrates. The mobile platform includes a 3D mobile platform, a servo tester, and fixtures, enabling the flexible end effector to move in 3D space. The pneumatic control system is composed of an Arduino Nano development board, an air pump, a pressure sensor, a solenoid valve, a buffer air bag, a DC motor speed control board, a step-down module, and a power supply; the PC terminal is equipped with an upper computer interface programmed based on LabVIEW, which can adjust the air pressure in real-time. The image-acquisition system is a GoPro high-definition camera, which can record the experimental process. The si*x*-axis force sensor has a range of 50 N, a resolution of 0.01 N, and a sampling rate of 2000 K/s, which meets the requirements of this experiment. It can collect force data in six dimensions in real-time and transmit it synchronously to the visualization interface of the PC terminal for storage. The target adhesion substrates of the experimental platform include rough planes (5-mesh, 10-mesh, 15-mesh, 20-mesh) in Figure 6A, smooth planes in Figure 6B, and rough curved surfaces (15-mesh rough curved surfaces with three curvature radii: 250 mm, 333 mm and 500 mm) in Figure 6C.

The substrate to be tested is fixed on the mobile platform with screws; the flexible bio-inspired end effector is fixed to the si*x*-axis force sensor with fixtures; the si*x*-axis force sensor is fixed on the mobile platform with an L-shaped connector. The flexible end effector is kept parallel to the surface to be tested, and the servo on the L-shaped connector is adjusted by the servo tester to control the contraction of the cable-driven system. The force sensor can measure forces and moments in six dimensions. When external tension or pressure acts on the sensor, the internal sensitive elements deform to generate weak electrical signals, which are amplified by the signal-amplification module and then converted into digital signals by the analog-to-digital converter in the data-acquisition card, enabling multi-channel acquisition. This experiment measures the tangential and normal adhesion forces of the flexible end effector during grasping.

## 4. Results

### 4.1. Axial Bending Performance of the Flexible Corrugated Telescopic Cavity

Alan T. Asbeck et al. proposed using a sweeping method to track the adhesion trajectory of claws on rough surfaces [34]. Without considering the bouncing phenomenon that may occur when claws slide, they found that claw tips do not adhere to all positions along the path; instead, they slide to find protrusions or depressions that allow adhesion. The final conclusions are drawn as follows: the smaller the contact angle between the claw and the surface, the more regions on the surface where adhesion is possible and the higher the friction force generated by the contact and loading of the claw on the surface. In addition, when the contact loading angle between the claw and the surface is in the range of not less than 45° and not more than 65°, the difference in the attachable area is insignificant; however, when the contact loading angle is not less than 80°, the attachable area decreases sharply.

To determine the claw installation angle of the flange buckle, a single flexible bio-inspired telescopic cavity was designed and fabricated. Its state under different air pressures was observed to study its bending performance, and the bending angle of the telescopic cavity under negative pressure was superimposed with the initial position of the claw to obtain a suitable contact loading angle for the claw during the operation of the flexible end effector, ensuring that the claw is in the optimal contact position for adapting to rough surfaces.

**Figure 7:**
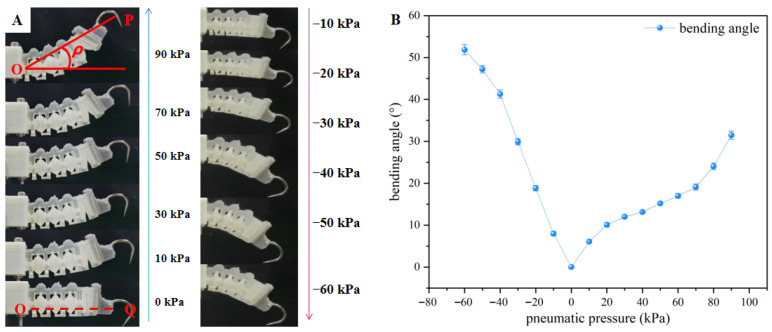
(**A**) Schematic diagram of bionic toe deformation under the action of positive and negative pressure; (**B**) curve of bionic toe bending angle variation under different air pressure conditions (N = 5).

The parameters of the telescopic cavity are defined as shown in Figure 7A. A horizontal line is drawn from the center point O of the air inlet of the telescopic cavity, intersecting the claw at point Q; the bending angle *ρ* of the flexible bio-inspired telescopic cavity is defined as the angle between the line OP and the horizontal line. A fixture was designed to fix the telescopic cavity; positive/negative pressure was applied to the flexible bio-inspired telescopic cavity via the pneumatic control system (limited by design requirements and the pneumatic system, the positive/negative pressure range is −60 to +90 kPa), yielding a comparison diagram of the telescopic cavity’s bending deformation under different air pressures. As shown in Figure 7B, within the negative pressure range of 0 to −60 kPa, the bending angle gradually increases. From 0 to −40 kPa, the bending angle increases approximately linearly; from −40 to −60 kPa, the growth rate slows down. At −40 kPa, the bending angle is 41°; at −30 kPa, it is 30°. In the positive pressure range of 0 to 90 kPa, the bending angle also shows an increasing trend: it grows rapidly from 0 to 20 kPa, slows down from 20 to 60 kPa, and accelerates again from 60 to 90 kPa. At 30 kPa, the bending angle is 12°; at 40 kPa, it is 13°. The two values are approximately equal.

### 4.2. Adhesion Performance of the Multi-Mechanism, Integrated Flexible End Effector on Smooth Surfaces

The test substrate of the multi-mechanism, integrated flexible end effector’s adhesion performance test platform was replaced with a smooth organic glass surface (Figure 6A). A stabilized DC power supply (5 V, 0.8 A) was set up and connected to the air source. First, positive pressure was inputted into the telescopic cavity, whose front end expanded, bent, and tilted upward, thereby avoiding interference from the front claws with the smooth surface. Then, the suction cup was connected to the negative-pressure air source; the mobile platform drove the end effector to move toward the tested substrate until the suction cup adsorbed to the acrylic plate. The mobile platform was adjusted to move in the reverse direction until the end effector detached from the smooth substrate (i.e., contact failure occurred); the peaks of tangential and normal forces during this process were recorded to evaluate the end effector’s adhesion capability on smooth surfaces. By adjusting the input negative pressure value, repeated tests were conducted to assess the impact of different pressures on adhesion.

**Figure 8:**
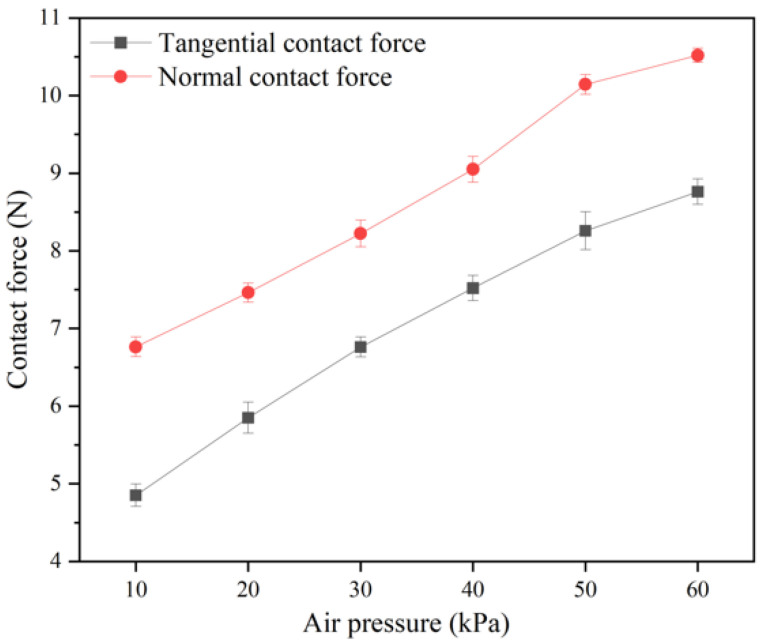
Curves of tangential and normal contact force variation of attachment end effector suction cup under different negative pressure conditions (N = 5).

As shown in Figure 8, for the suction cup of the end effector (fabricated from Silicon 40A material), in the absence of significant external pre-pressure, the normal and tangential forces increased approximately linearly with an increasing negative pressure. At a negative pressure of 10 kPa, the suction cup generated a normal adsorption force of 6.78 N and a tangential adsorption force of 4.84 N; at 40 kPa, it generated 9.06 N (normal) and 7.51 N (tangential); at 60 kPa, it generated 10.51 N (normal) and 8.70 N (tangential). The normal and tangential adsorption forces approximately satisfy Figure S7 (Section S2 in Supplementary Materials). Thus, under a negative pressure of 60 kPa, the suction cup exhibited a normal adsorption force of 10.51 N and a tangential adsorption force of 8.70 N on the smooth acrylic plate, which represents increases of approximately 55% and 76% compared to the values at 10 kPa. This indicates that the suction cup has good adsorption performance on smooth acrylic plates.

### 4.3. Adaptability of the End Effector to Rough Surfaces

The test substrate of the multi-mechanism, integrated flexible end effector’s adhesion performance test platform was replaced with rough planes/curved surfaces of different meshes (Figure 6B,C). The 3D mobile platform drove the end effector to descend slowly and uniformly in the vertical direction to contact the rough substrate to be tested. Negative pressure was applied to the flexible telescopic cavity to drive the claws to bend inward, thereby making contact with the rough substrate. The end effector was then moved uniformly 15 mm in the horizontal direction at a speed of 2 mm/s.

During the movement process, the claw searches for an attachment point on the surface. When it comes into contact with a particle protrusion, the claw embeds itself into the protrusion and stops moving. Then, the attachment device is driven by the mobile platform to continuously change the adhesion angle, and the tangential adhesion force increases gradually until the claw is pulled out of the hooking state. After that, the attachment device continues to move to search for the next attachment point.

After moving 15 mm, the horizontal movement and negative pressure supply stop, and the mobile platform drives the attachment device to move upward uniformly along the vertical direction. Then, the platform moves upward by 40 mm to reset the claw-attachment device to its original position. The above operation is repeated more than five times, and the peak force during this process is recorded to evaluate the adhesion capacity of the attachment device to the target substrate surface. For the normal force experiment, when the flexible attachment device moves downward and comes into contact with the rough curved surface initially, the platform moves upward along the vertical direction after the negative pressure input stabilizes to obtain the normal force.

**Figure 9:**
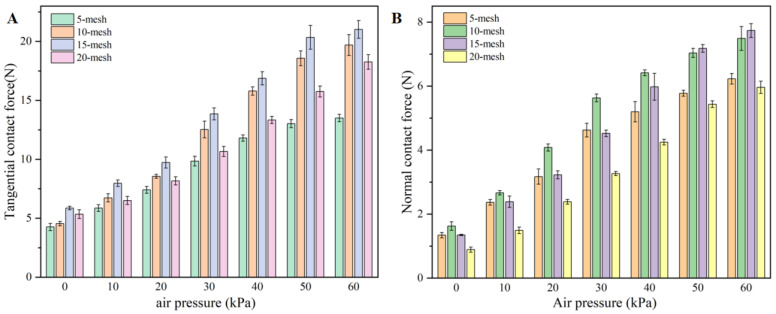
Curves of contact force variation of flexible attachment device on surfaces of various mesh sizes under different negative pressure conditions. (**A**): Tangential contact force; (**B**): normal contact force.

Figure 9 shows the variation of tangential and normal adhesion forces of the flexible end effector under different negative pressures on rough planes of different meshes. It can be seen that with the increase of negative pressure, the tangential adhesion force and normal adhesion force exhibit an overall trend of nonlinear growth. The specific characteristics are as follows: the force increases approximately linearly in the range of 0–20 kPa, then rises sharply in the range of 20–40 kPa, while the growth rate decreases and tends to be constant again in the range of 40–60 kPa, showing a relatively stable state. Taking the adhesion experiment of the flexible attachment device on a 15-mesh rough surface as an example, when the negative pressure ranges from 0 to 20 kPa, the tangential adhesion force increases from 5.86 N at 0 kPa to 9.47 N at −20 kPa, and the normal adhesion force increases from 1.34 N at 0 kPa to 3.22 N at −20 kPa. In this stage, for every 10 kPa increase in negative pressure, the tangential adhesion force increases by approximately 1.2 N and the normal adhesion force increases by approximately 0.94 N, with a relatively low growth rate. When the negative pressure ranges from 20 to 40 kPa, the tangential adhesion force reaches 16.86 N at −40 kPa and the normal adhesion force reaches 5.98 N at −40 kPa. In this stage, for every 10 kPa increase in negative pressure, the tangential adhesion force increases by approximately 3.7 N and the normal adhesion force increases by approximately 1.38 N, with a higher growth rate. When the negative pressure ranges from 40 to 60 kPa, the tangential adhesion force reaches 21.02 N at −60 kPa and the normal adhesion force reaches 7.74 N at −60 kPa. In this stage, for every 10 kPa increase in negative pressure, the tangential adhesion force increases by approximately 2.1 N and the normal adhesion force increases by approximately 0.88 N, with a slowed growth rate.

From a horizontal comparison, the tangential adhesion force of the claw on the 15-mesh rough surface is generally stronger than that on the other three mesh rough surfaces and performs the worst on the 5-mesh rough surface. On the 5-mesh rough surface, the tangential adhesion force of the claw is only 11.80 N at −40 kPa and only 13.5 N at −60 kPa. In contrast, on the 15-mesh rough surface, the tangential adhesion force of the claw is 16.86 N at −40 kPa and 21.02 N at −60 kPa, with a large difference of 5.06 N and 7.52 N, respectively. This indicates that the claw achieves the best and most significant adhesion performance on the 15-mesh rough surface. The particle protrusion size of the 15-mesh surface is moderate for the claw tip diameter, providing a larger surface area with attachable protrusion particles. A short displacement slide enables the claw to easily hook the protrusion particles and generate a relatively large tangential adhesion force. In contrast, although the protrusion particles on the 5-mesh rough surface are relatively large, the claw cannot ensure stable and good contact during short displacements on the surface, resulting in a lower tangential adhesion force compared with that on other mesh rough surfaces.

In contrast, the normal adhesion force of the claw on the 10-mesh rough surface is generally stronger than that on the other three mesh rough surfaces. The performance on the 15-mesh surface is comparable to that on the 10-mesh surface, while the performance on the 20-mesh surface is the worst. On the 20-mesh rough surface, the tangential adhesion force of the claw is only 4.24 N at −40 kPa and only 5.96 N at −60 kPa. In contrast, on the 10-mesh rough surface, the tangential adhesion force of the claw is 6.41 N at −40 kPa and 7.50 N at −60 kPa, with a large difference of 2.17 N and 1.54 N, respectively. The protrusion particles on the 5-mesh surface are relatively large. Driven by the deformation of the telescopic cavity, although the initial normal force of the claw is relatively large, the irregularity of the surface structure of the protrusion particles increases. After the deformation of the flexible attachment device, the locking stability between the claw and the protrusion particles is weaker than that on the 10-mesh or 15-mesh surface, resulting in a poor adhesion environment and a tendency to fall into a false hooking state. Therefore, the normal force at −60 kPa is lower than that on the 10-mesh and 15-mesh surfaces. On the 20-mesh rough surface, the protrusion particles are relatively small. The dense arrangement of small-sized particles reduces the attachable area for the claw and shortens the slidable distance provided by the small protrusion particles, making it difficult to form a stable hooking state. Thus, the growth amplitude of the normal force is smaller than that on the 10-mesh and 15-mesh surfaces.

**Figure 10:**
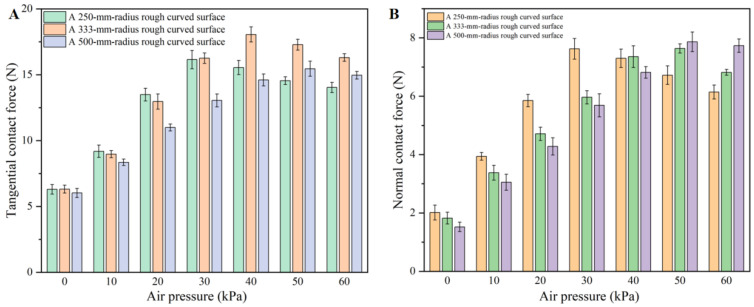
Curves of contact force variation of flexible attachment device on rough curved surfaces with various curvature radii under different negative pressure conditions. (**A**): Tangential adhesion force; (**B**): normal adhesion force.

The relationship between the tangential and normal adhesion forces generated by the flexible attachment device under different negative pressures on rough curved surfaces with different curvature radii is shown in Figure 10. It can be seen that with the increase in negative pressure, the tangential adhesion force shows a trend of first increasing and then slowly decreasing. For example, when the flexible attachment device works on the rough curved surface with a curvature radius of 333 mm, the tangential adhesion force increases rapidly from 6.31 N at 0 kPa to 18.05 N at −40 kPa with a large increment of 11.74 N when the negative pressure ranges from 0 to 40 kPa. However, when the negative pressure ranges from 40 to 60 kPa, the tangential adhesion force decreases slowly, with a slight reduction from 18.05 N to 16.30 N at −60 kPa and a slowed decreasing rate of 1.75 N.

When the flexible attachment device realizes enveloping adhesion on the rough curved surface with a curvature radius of 250 mm, the normal adhesion force increases by 5.61 N from 2.01 N at 0 kPa to 7.62 N at −30 kPa and then decreases slowly by 1.48 N to 6.14 N at −60 kPa. When the curvature radius is 333 mm, the normal adhesion force increases by 5.79 N from 1.82 N at 0 kPa to 7.61 N at −40 kPa and then decreases slowly by 0.79 N to 6.82 N at −60 kPa. When the curvature radius is 500 mm, the normal adhesion force increases by 6.34 N from 1.52 N at 0 kPa to 7.86 N at −50 kPa and then decreases slowly by 0.13 N to 7.73 N at −60 kPa.

Among the three curvature radii, the surface with a curvature radius of 333 mm shows a slightly higher peak tangential adhesion force, while the peak normal adhesion forces are comparable. We note that this difference may be related to how the deformation state and contact configuration evolve under different curvatures

### 4.4. Robot Climbing Experiment Verification on Different Target Surfaces

Climbing experiments were conducted to test the robot’s climbing performance on cross-scale roughness surfaces, verifying whether the designed flexible end effector enables the robot to achieve the expected grasping and detachment behaviors. Four tests were performed: climbing on a 90° vertical rough plane, a 90° vertical smooth plane, a rough curved surface, and a smooth arc surface. During climbing, the robot does not explicitly classify the surface texture; instead, surface changes are inferred through real-time vacuum pressure feedback. Due to differences in sealing performance, smooth surfaces enable a higher and more stable negative pressure, whereas rough surfaces lead to a reduced achievable vacuum, which triggers switching from suction-dominated adhesion to the claw-interlocking mode. This feedback-driven switching strategy follows the robust disturbance-aware control principles commonly adopted in small-scale robotic systems [35,36].

**Figure 11:**
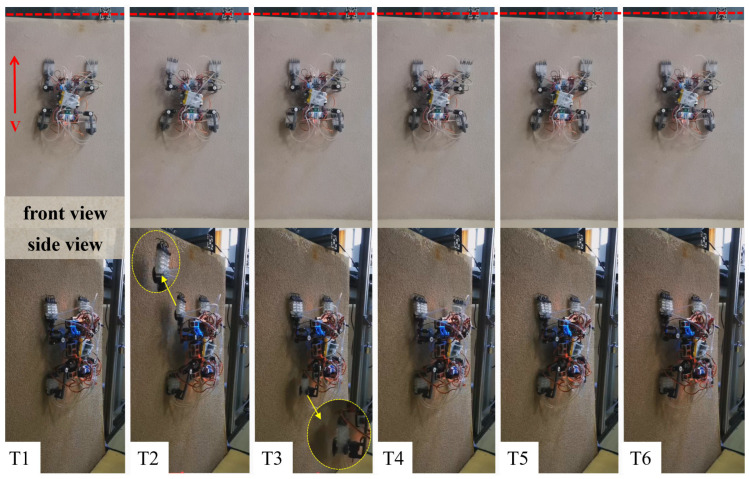
Diagram of robot climbing process on vertical rough flat surface. (Note: T1: initial position; T2: lift the left front leg, move forward and adsorb; T3: lift the left rear leg, move forward and adsorb; T4: lift the right front leg, move forward and adsorb; T5: lift the right rear leg, move forward and adsorb; T6: completion of forward movement).

The robot achieved stable climbing on a 90° vertical rough surface (20-mesh roughness), with the specific climbing process shown in Figure 11.

**Figure 12:**
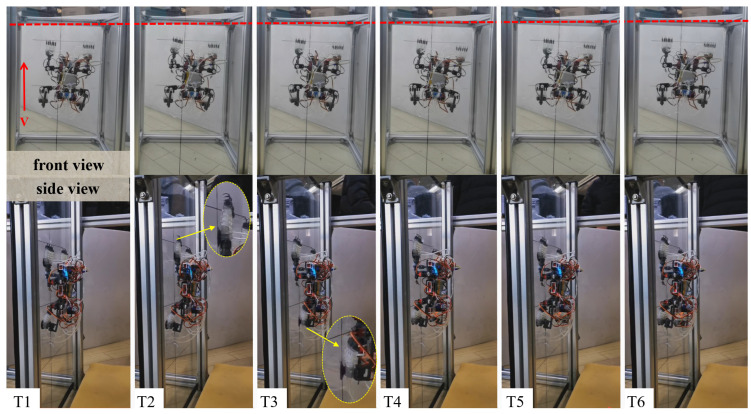
Diagram of robot climbing process on vertical smooth flat surface. (Note: T1: initial position; T2: lift the left front leg, move forward and adsorb; T3: lift the left rear leg, move forward and adsorb; T4: lift the right front leg, move forward and adsorb; T5: lift the right rear leg, move forward and adsorb; T6: completion of forward movement).

The robot also achieved stable climbing on a 90° vertical smooth surface (smooth acrylic plate), with the specific climbing process shown in Figure 12.

**Figure 13:**
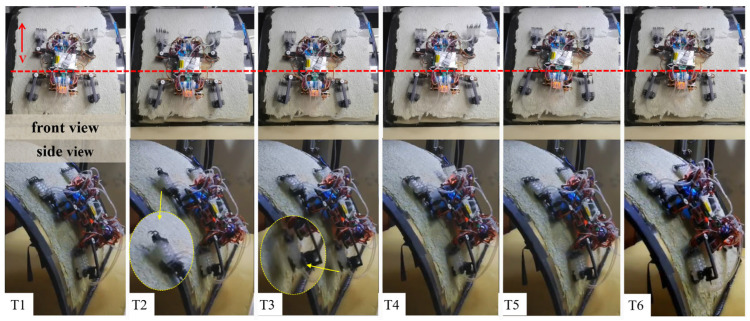
Diagram of robot climbing process on rough curved surface. (Note: T1: initial position; T2: lift the left front leg, move forward and adsorb; T3: lift the left rear leg, move forward and adsorb; T4: lift the right front leg, move forward and adsorb; T5: lift the right rear leg, move forward and adsorb; T6: completion of forward movement).

The robot achieved stable climbing on a rough curved surface (10-mesh roughness), with the specific climbing process shown in Figure 13.

**Figure 14:**
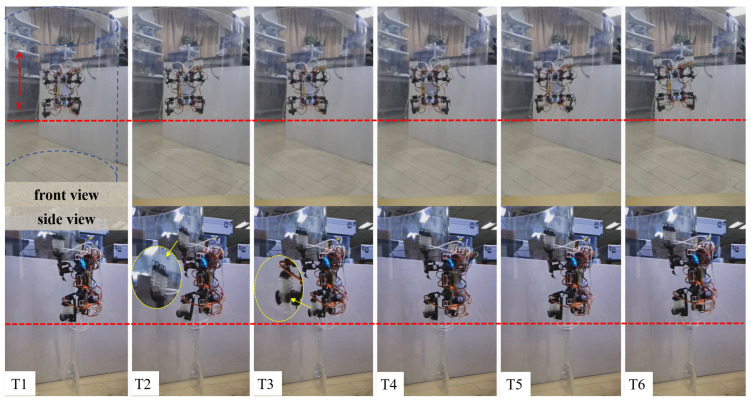
Diagram of robot climbing process on smooth curved surface. (Note: T1: initial position; T2: lift the left front leg, move forward and adsorb; T3: lift the left rear leg, move forward and adsorb; T4: lift the right front leg, move forward and adsorb; T5: lift the right rear leg, move forward and adsorb; T6: completion of forward movement).

The robot achieved stable climbing on a smooth arc surface (smooth acrylic plate), with the specific climbing process shown in Figure 14.

In summary, the robot can climb not only on 90° vertical rough and smooth planes but also on rough and smooth arc surfaces with stable adhesion and detachment. Slight deviations from the vertical movement direction occasionally occurred during the test, which is normal. The main reasons are uncontrolled robot operation and irregular actual surface features (especially on rough surfaces), leading to asymmetric adhesion of the flexible end effector and slight changes in the head orientation. These deviations accumulate over time, eventually causing the robot’s movement to slightly deviate from the intended direction.

## 5. Discussion

In nature, the tarsal claws, micro-spines, or barbs of many organisms such as beetles and locusts are independent of each other. This facilitates the uniform distribution of loads and improves the adhesion probability and efficiency between the claws and rough surfaces during dynamic contact. Therefore, this paper develops a flexible telescopic chamber as the driving component, whose flexibility enhances the adaptability of the attachment device to surface morphologies. Whether for planar or curved surface adhesion, the front-end claws can generate approximately 5 N of tangential adhesion force and 1 N of normal adhesion force under a preload contact force of about 0.4 N without negative pressure input. This indicates that the attachment device still possesses basic adhesion capability under zero-negative-pressure conditions, and the load in this stage is mainly borne by the rigid structure of the claws themselves.

With the increase of negative pressure, the adhesion performance presents obvious stage-specific laws. In the low negative pressure range, due to the small air input of the telescopic chamber and the low internal structural stress level, the overall stiffness and torsion resistance are limited. The effective interlocking and stable contact between the claws and rough particles are insufficient, so the tangential and normal adhesion forces only increase slowly with the increase in negative pressure. This stiffness-mediated enhancement of adhesion is consistent with previous findings, where increasing the flexural rigidity of soft actuators was shown to improve adhesion performance on curved surfaces [37].

When the negative pressure exceeds about 30 kPa, the adhesion force increases significantly. On the one hand, the negative pressure increases the internal stress of the telescopic chamber and makes the structure tend to be compact, thereby improving the torsional stiffness and tensile stiffness and suppressing the slight rotation and disturbance during the adhesion process. On the other hand, the negative pressure increases the bending degree of the telescopic cavity, and the contact angle and attachable area of the claws expand accordingly, which is more conducive to the claws forming interlocks in the gaps or corners of the protruding particles. Therefore, the adhesion force increases more significantly in this stage.

When the negative pressure exceeds ~50 kPa, the measured adhesion force shows a reduced growth rate and, in some cases, a slight decrease. This suggests that further increasing the negative pressure does not continuously improve the effective interlocking condition. A possible explanation is that the deformation/contact configuration approaches a limiting state at high negative pressures. Notably, the dispersion (error bars) increases at higher negative pressures, indicating increased variability in the measurements.

From the longitudinal trend, the tangential and normal adhesion forces on rough planes with different mesh numbers generally show the variation law of approximately linear growth at low negative pressure, a sharp rise at medium negative pressure, and a growth rate decrease and stabilization at high negative pressure with the increase in negative pressure. From the horizontal comparison, the tangential adhesion force of the claws on the 15-mesh rough surface is generally stronger, while the normal adhesion force on the 10-mesh and 15-mesh rough surfaces is generally higher than that on surfaces with other mesh numbers.

Under the condition of rough curved surfaces, the adhesion force shows a peak characteristic of first increasing and then slowly decreasing with the change in negative pressure. When the negative pressure increases from 0 to about 40 kPa, the bending deformation of the telescopic chamber gradually increases, the enveloping and fitting ability is enhanced, and the structural stiffness is improved at the same time, which is conducive to the claws forming a more stable interlocking contact. Therefore, the tangential and normal adhesion forces reach an optimal level.

When the negative pressure exceeds ~40 kPa, the adhesion force shows a mild decline after the peak. This suggests that an optimal negative pressure exists for the rough surface condition, and further pressurization does not continuously enhance the effective contact/interlocking. A possible explanation is that the contact/interlocking becomes more localized at higher deformation levels, reducing the effective contribution to tangential resistance; however, this mechanism is not directly resolved by the present force-only measurements.

The abnormal claw adhesion observed in the experiment is jointly related to the attachment device itself and the characteristics of the rough surface. For the attachment device itself, the flexible structure enhances its morphological adaptability, but insufficient torsional stiffness may trigger slight rotation and rebound under low negative pressure or uneven local loading. These issues cause disturbances or even slight bounces in the hooking of the claw tips, thereby increasing the probability of detachment. In addition, if the strength of the claw tips is insufficient, there is a risk of plastic failure under repeated high loads.

The experimental observations suggest that the claw mechanism, suction-based adhesion, cable-driven adjustment, and pressure-induced deformation operate cooperatively, with different mechanisms becoming dominant under different surface conditions. For the rough surface, individual protruding particles fall off after the experiment, indicating that insufficient bonding strength between the particles and the substrate reduces the reliability of the interlocking objects. Negative pressure exerts a dual regulatory effect on the deformation and equivalent stiffness of the telescopic chamber: the medium negative pressure range can simultaneously improve the fitting performance and stability, which is the key to achieving better adhesion performance. In contrast, excessively high negative pressure may induce geometric mismatch and contact degradation, leading to limited or even decreased adhesion force gain. Therefore, the working negative pressure of the attachment device should be selected according to the wall roughness and curvature conditions to achieve a balance among fitting ability, structural stiffness, and interlocking effectiveness.

Vacuum suction-cup-based adhesion systems exhibit stable performance on smooth surfaces but are highly sensitive to sealing integrity. They are prone to leakage and failure when facing rough, porous, or gap-containing surfaces [3,4]. In contrast, claw-interlocking adhesion systems possess strong load-bearing capacity on rough surfaces but are limited by the availability of interlockable surface features, making them difficult to work effectively on smooth or low-roughness surfaces [6,7]. Trans-surface tasks usually rely on the juxtaposition and switching of two sets of end effectors or multiple adhesion modules. Many multi-modal adhesion/climbing systems extend the surface applicability by physically combining and mechanically switching among multiple attachment modules (e.g., spines/adhesives plus suction), which typically introduces additional actuators, interfaces, and housings, thereby increasing the system weight and packaging volume. A representative wall-climbing robot integrates spine wheels, adhesive belts, and an eddy suction cup, illustrating the need for multi-module integration and associated onboard hardware/space allocation in such designs [15]. Related suction/adhesion solutions that rely on added enabling components (e.g., magnetically controlled/switchable mechanisms) further imply extra mass/volume for actuation and packaging [11], while multimodal adhesion–suction end-effectors designed for manipulation likewise highlight the integration/complexity trade-off when combining multiple modalities within one device [38]. In addition, mechanically guided or sensorized suction cups require extra structural parts and embedded components, which also adds to the end-effector volume and weight budget [39]. By contrast, the proposed adhesion device achieves stable attachment on two typical surface types—smooth surfaces (Ra < 0.1 μm) and rough surfaces (Ra > 1 μm)—without additional adhesion module stacking. Negative pressure is utilized to regulate the deformation and equivalent stiffness of the telescopic chamber, while cable-driven actuation provides attitude and load adjustment. Related studies on multi-sensor fusion and contribution evaluations in teleoperated robotic systems provide useful perspectives for understanding the coupled influence of pressure regulation, cable-driven adjustment, and structural compliance in biomimetic adhesion systems [40]. This enables the system to obtain more stable adhesion in the medium negative pressure range. Mechanism complementarity realizes trans-surface adaptability, which improves the adhesion continuity and safety margin of trans-surface tasks under the premise of controllable system complexity.

## 6. Conclusions

To address the issue that a single adhesion mechanism is prone to leakage failure or insufficient interlocking on cross-roughness walls, leading to climbing interruption, this paper proposes and develops a flexible multi-mechanism coupled attachment device driven by a hybrid pneumatic-cable system. The device integrates claw-interlocking adhesion and negative pressure adsorption within the same end effector and uses pressure as a unified state variable to regulate the contact morphology and load-bearing path, thereby achieving switchable rough-surface adhesion and smooth-surface adsorption.

Experiments on rough flat surfaces demonstrate that the adhesion force exhibits a segmented nonlinear growth trend with increasing negative pressure. Taking the 15-mesh surface as an example, the tangential adhesion force increases from 5.86 N (0 kPa) to 16.86 N (−40 kPa) and reaches 21.02 N at −60 kPa; the normal adhesion force rises from 1.34 N to 5.98 N (−40 kPa) and peaks at 7.74 N at −60 kPa. A horizontal comparison indicates that the 15-mesh surface yields the optimal tangential performance (7.52 N higher than that of the 5-mesh surface at −60 kPa), while the normal adhesion force is superior on the 10–15-mesh surfaces and weakest on the 20-mesh surface (only 5.96 N at −60 kPa). These results verify that the claw performance is constrained by the scale and density of interlockable surface features.

Experiments on rough curved surfaces reveal a distinct optimal pressure range. Taking the curved surface with a curvature radius of 333 mm as an example, the tangential adhesion force increases from 6.31 N (0 kPa) to 18.05 N (−40 kPa) and then decreases to 16.30 N at −60 kPa; the normal adhesion force peaks at approximately 7.6–7.9 N (occurring at −30/−40/−50 kPa, depending on the curvature radius).

After integrating the attachment device into the robot, stable adhesion and detachment are achieved on 90° vertical rough/smooth flat surfaces and rough/smooth curved surfaces. This validates the feasibility of continuous cross-surface climbing from smooth to rough surfaces without end-effector replacement. Without stacking multiple adhesion systems, switchable adhesion and continuous climbing across variable-roughness walls are realized. Quantitative performance boundaries and recommended pressure ranges under different roughness and curvature conditions are provided, which offer experimental evidence and engineering guidance for the lightweight, integrated and controllable-switch design of end-effector attachments for multi-surface adaptive climbing robots. Future work will (i) evaluate the wear resistance and fatigue life of the telescopic cavity under long-term rough-surface friction, with possible material modification and protective/wear-resistant coatings; (ii) perform ablation/decoupling experiments to quantify the individual contributions of each adhesion mechanism and optimize their coupling; and (iii) conduct robot-level tests across diverse surfaces and loads to benchmark climbing speed, stability, and energy efficiency.

## Figures and Tables

**Table 1:** Parameter selection for the front claw.

Name	Parameter Value
Angle $\alpha_{k}$ between the claw and the bottom of the flange buckle in the initial position	70°
Distance $H_{k}$ from the claw tip to the bottom of the claw flange buckle	7 mm
Claw diameter $d_{k}$	1 mm
Claw width $P_{k}$	12 mm

Note: the angle $\alpha_{k}$ of the front claw is selected based on experiments and existing theories, using the ideal contact angle between the claw and the surface as the corresponding parameter.

**Table 2:** Parameter selection of the telescopic unit.

Name	Parameter Value
Shape	Sloped flat type
Wall thickness $H_{a}$	0.4 mm
Wall thickness $H_{b}$	1
Included angle β	36°
Included angle α	108°

Note: the telescopic unit is based on finite element simulation analysis, and the structural parameters corresponding to the maximum adhesion force are selected.

**Table 3:** Reserved length for cable-driven system attaching to surfaces with different curvatures.

Attached Surface	Reserved Cable Length (mm)
Curved surface with a radius of 250 mm	−7 to −6
Curved surface with a radius of 330 mm	−5 to −4
Curved surface with a radius of 500 mm	−3 to −2
Rough flat surface	0
Smooth flat surface	>5

## Data Availability

The original contributions presented in this study are included in the article/Supplementary Materials. Further inquiries can be directed to the corresponding authors.

## Supplementary Materials

The following supporting information can be downloaded at: https://www.mdpi.com/article/10.3390/biomimetics11020140/s1, Figure S1: Structural parameter analysis of the telescopic unit (influence of contour shape). Figure S2: Simulation analysis of telescopic cavity wall thickness. Figure S3: Influence of length–diameter ratio (W/L ratio) on normal tensile force. Figure S4: Influence of taper angle under fixed annular cross-section conditions. Figure S5: Influence of taper angle under fixed air inlet/outlet conditions. Figure S6: Contact state and force analysis of the suction cup. Figure S7: Friction coefficient test of the silicone suction cup on an acrylic surface. Section S1: Structural Parameter Analysis of the Telescopic Unit. Section S2 Structural Design and Friction Coefficient of the Suction Cup.
