# Supplementary material for: Pneumatic–Cable-Hybrid-Driven Multi-Mechanism End Effector and Cross-Surface Validation
Source: Biomimetics (Basel). 2026 Feb 12;11(2):140. doi: 10.3390/biomimetics11020140 (PMC12938715; doi:10.3390/biomimetics11020140)
Supplement: Supplementary file 1 [file biomimetics-11-00140-s001.zip › biomimetics-4134726-supplementary.pdf]

Supplementary

S1 Structural Parameter Analysis of the Telescopic Unit

S1.1 Shape of the Telescopic Cavity

The telescopic cavity is the basic unit for realizing the function of the bionic toe, responsible for the bending deformation of the flexible attachment device (Fig. S1A). The outer contour of the telescopic cavity affects the deformation state and load-bearing mode of the entire flexible palm, thereby determining the deformation trend of the bionic toe. In this study, three types of telescopic cavity structures are established: outer convex type, inclined flat type, and inner concave type (Fig. S1B). Except for the different contour shapes, all other parameters are consistent. The diameter of the largest outer circle in the middle of the telescopic cavity is 9 mm, the overall transverse width of the cavity is 5 mm, the initial wall thickness is 0.3 mm, the diameter of the air inlets/outlets at both ends is 2 mm, the width and height of the strain constraint layers at both ends are 5 mm and 11 mm respectively, and the height and width of the tendon sheath at the top of the cavity are set to 2 mm and 3.2 mm. The annular perimeter parallel to the inner wall of the cavity at 1/4 of the horizontal axis is defined as the left neck, and that at 3/4 of the horizontal axis is defined as the right neck.

The telescopic cavity model was built in SolidWorks and directly imported into ABAQUS for preprocessing. The selected material is a hyperelastic materials, defined as Elastic 50A with a density of 1e-09, and the applicable strain energy model is the Mooney–Rivlin mode (strain energy: $W = C_{10}(I_1 - 3) + C_{01}(I_2 - 3) + \frac{1}{d}(J-1)^2$, C_{10} = 0.4138， C_{01} = 0.1034， d = 0.2[Error! Reference source not found.]).

Considering the existing robot body and energy supply capacity of the team, the maximum positive pressure input of the flexible bionic adhesion palm does not exceed 90 kPa, and the maximum negative pressure input does not exceed 60 kPa. A uniform pressure of -30 kPa was applied to the entire inner wall of the telescopic cavity.

For boundary conditions:

The entire leftmost surface was fixed, with six degrees of freedom constrained (displacement/rotation restricted in all directions). The center of the air inlet/outlet on the right cavity was also constrained with six degrees of freedom.

Two analysis steps were set:

Step 1: Fix the leftmost cavity wall, apply a uniform load of -30 kPa to the inner surface of the telescopic cavity, and set all six degrees of freedom (displacement/rotation) of the right air inlet/outlet center to 0. This step simulates the process where the strain constraint layers on both sides are not driven by the middle bellows to undergo large displacement deformation when negative pressure is just introduced into the air cavity in the actual working environment.

Step 2: Release the X-direction (negative direction) displacement constraint of the right air inlet/outlet center and set a displacement of 1 mm in the negative X-direction, while keeping other settings unchanged. This step simulates the process where the middle bellows contracts inward under negative pressure, driving the right strain constraint layer to move toward the left.

For field variable output: The tensile force, stress, and strain of the right air inlet/outlet center during the negative-pressure contraction of the bellows were output.

As shown in Fig. S1C-D, the telescopic cavity is restricted and fixed by the strain constraint layers on both sides, and inward contraction deformation starts from the left neck and right neck. Stress first concentrates at these two positions and the middle symmetry plane, while the displacement and stress between the necks on both sides and the middle symmetry plane are relatively small. When the X-direction displacement constraint of the right air inlet/outlet center is released, the telescopic cavity does not undergo excessive torsion due to low radial stiffness, and the expected deformation under negative pressure is achieved.

The negative-pressure deformation process of the inner concave telescopic cavity is roughly consistent with that of the inclined flat type. The inclined flat type takes slightly longer to adjust torsion and swing, but its maximum stress value is smaller than that of the inner concave type. However, the negative-pressure deformation process of the outer convex telescopic cavity differs

significantly from the other two types. After the left and right necks start to bend toward the middle, the radial stiffness of the structure is too low, leading to excessive annular torsion at the necks, and the structure is radially crushed by external air pressure at the symmetry plane, thus failing to achieve the expected deformation.

The inner concave and inclined flat structures have little impact on the negative-pressure contraction process, and their deformation processes are similar. Under -30 kPa air pressure, the normal forces generated at the fixed end of the cavity are 0.66 N and 0.62 N, respectively (with a small difference), while the outer convex type cannot complete the contraction deformation normally under -30 kPa air pressure. By comprehensively balancing the deformation trend of the telescopic cavity and the stability of subsequent manufacturing, the inclined flat type is selected as the contour shape of the telescopic cavity.

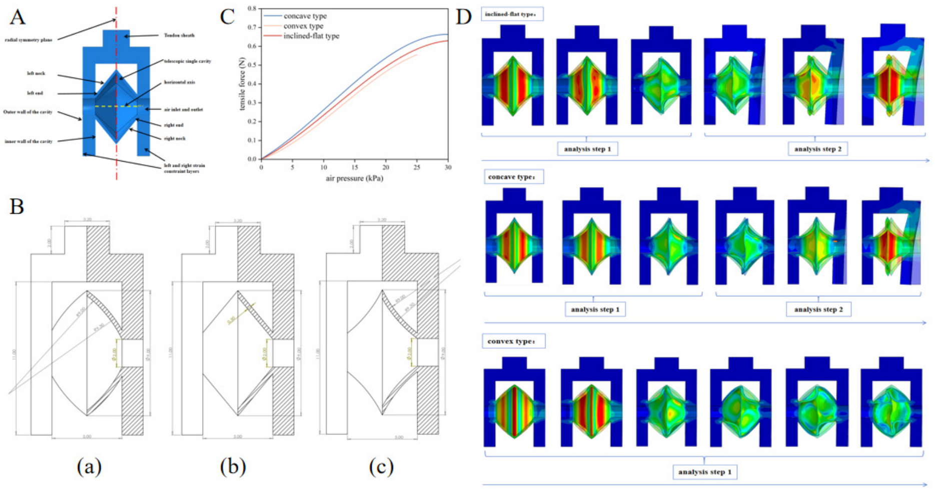

Fig. 2.2 S1A: Structure of the telescopic cavity; B: structural parameters of telescopic cavities with three contour shapes; C: normal tensile force curves of telescopic cavities with three contour shapes under different negative pressures; D: simulated deformation states of telescopic cavities with different shapes

S1.2 Simulation Analysis and Influence of Telescopic Cavity Wall Thickness

The wall thickness of the telescopic cavity is also a key factor affecting its deformation and normal tensile force (S2A). The wall thickness H_a was set to 0.3 mm, 0.4 mm, 0.5 mm, 0.6 mm, and 0.7 mm, respectively. Except for the wall

thickness, the inclined flat contour shape and other settings were retained.

Taking the telescopic cavity with a wall thickness of 0.4 mm under -50 kPa as an example (S2B), its simulation deformation process is similar to that of the inclined flat type in the previous section. A larger wall thickness provides greater radial stiffness, which can restrict unnecessary radial deformation and maintain the basic shape. For the wall thickness of 0.3 mm, the telescopic cavity is crushed under -40 kPa and -50 kPa: torsion occurs at the beginning of the first analysis step. Due to the small wall thickness (low radial stiffness), the radial deformation is too large to resist the resultant radial force from the external atmospheric pressure, leading to non-convergence.

As shown in Fig. S2C, under the same negative pressure, the normal tensile force at the right end of the telescopic cavity gradually decreases as the wall thickness increases from 0.4 mm to 0.7 mm. At -50 kPa, the normal tensile force decreases by 0.13 N (from 0.96 N to 0.83 N) when the wall thickness increases from 0.4 mm to 0.5 mm; at -40 kPa, the tensile force decreases by 0.16 N (from 0.80 N to 0.64 N) for the same wall thickness change. Although the structure with a wall thickness of 0.3 mm cannot provide sufficient radial stiffness under high negative pressure to support the designed deformation process of the telescopic cavity, considering the normal tensile force performance and the requirements for axial/radial stiffness, the wall thickness of the telescopic cavity is set to 0.4 mm.

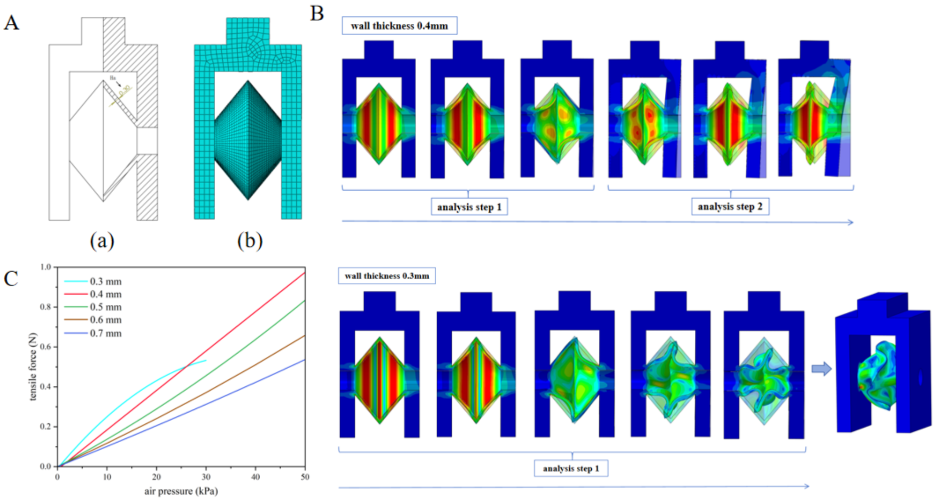

Fig. S2: A Telescopic cavity diagram with wall thickness as a parameter (Note: a Identification of wall thickness in the telescopic cavity; b Mesh division diagram of the telescopic cavity with wall thickness as a parameter); B simulation deformation processes

of telescopic cavities with two wall thicknesses under -50 kPa (Note: a Deformation of the telescopic cavity with 0.4 mm wall thickness; b Deformation of the telescopic cavity with 0.3 mm wall thickness); C normal tensile force curves of telescopic cavities with different wall thicknesses under different negative pressures

S1.3 Simulation Analysis and Influence of Length–Diameter Ratio of the Telescopic Cavity

When negative pressure is inputted, the outer contour of the telescopic cavity has a significant impact on the overall deformation. If a section is taken along the middle symmetry plane, the resulting cross-section is not an annular shape but a straight-slot annular shape. Two parameters define the shape of the straight slot: slot length and slot width.

This section explores the influence of the ratio H_b (slot width to slot length) on the normal tensile force at the right end of the telescopic cavity when its cross-section is a straight slot (S3A). To follow the single-variable principle, the long side of the telescopic cavity cross-section (i.e., the slot length of the straight slot) is fixed at 10 mm, and the slot width is set to 10 mm, 9 mm, 8 mm, 7 mm, and 6 mm, respectively. Thus, the width-to-length ratios (W/L ratios) are set to 1, 0.9, 0.8, 0.7, and 0.6. In addition, the shapes of the left and right end faces of the telescopic cavity are projected onto the end faces at a 45°angle based on the cross-section. The thickness is 0.4 mm (determined in the previous section), and other parameters remain consistent.

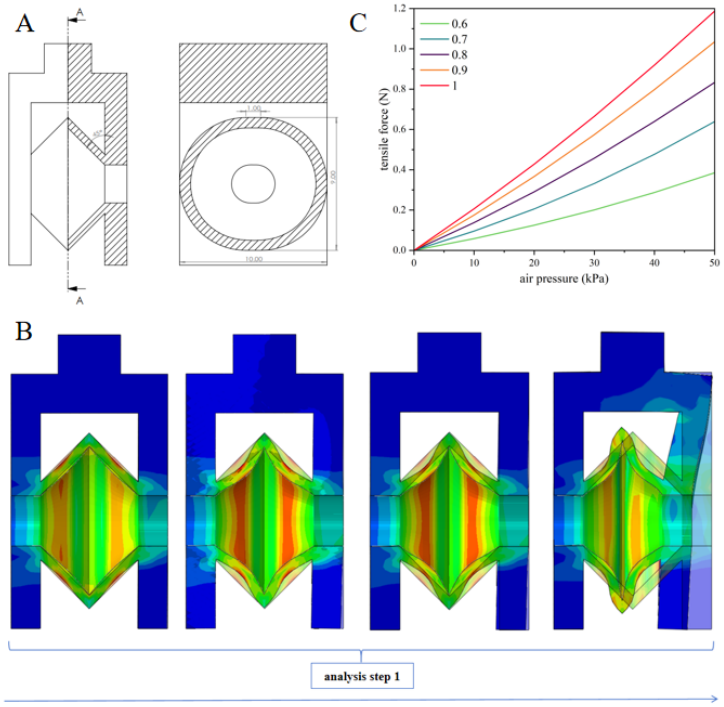

Fig. S3: A Identification of W/L ratio in the telescopic cavity; B simulation deformation process of the telescopic cavity with W/L ratio = 0.9 under -50 kPa; C normal tensile force curves of telescopic cavities with different W/L ratios under various negative pressures.

As shown in Fig. S3B, the simulation results do not exhibit non-convergence phenomena (as seen in the previous two sections). Under negative pressure, stress concentration first occurs at the left and right necks, and the outer wall of the entire telescopic cavity contracts inward under atmospheric pressure. However, no torsion-induced significant radial displacement is observed, indicating that the telescopic cavity has sufficient radial stiffness under this parameter setting to achieve stable contraction deformation under negative pressure. Under a fixed pressure from the pneumatic system, as the W/L ratio increases from 0.6 to 1, the outer surface area of the telescopic cavity increases accordingly. Therefore, under the same negative pressure, the atmospheric pressure acting on the outer surface of the telescopic cavity increases, ultimately driving the cavity to contract inward and generate a corresponding tensile force on the right end face when the left end face is fixed. As shown in Fig. S3C, the normal tensile force at the right end face gradually increases as the W/L ratio of the telescopic cavity increases. At a W/L ratio of 0.6, the tensile forces under -50 kPa and -40 kPa are 0.38 N and 0.29 N, respectively; at a W/L ratio of 1, the tensile forces under -50 kPa and -40 kPa are

1.19 N and 0.92 N, respectively—representing increases of 0.81 N and 0.63 N. Moreover, as the W/L ratio increases, the growth trend of the normal tensile force becomes more pronounced at higher negative pressures: at a W/L ratio of 0.6, the tensile force only increases by 0.33 N when the negative pressure rises from 10 kPa to 50 kPa; at a W/L ratio of 1, this increase reaches 0.98 N. Based on the above analysis, the tensile force effect is optimal when the W/L ratio of the telescopic cavity is 1 (i.e., the cross-section is annular). Therefore, the cross-section of the middle symmetry plane of the telescopic cavity is finally determined to be annular (instead of straight-slot annular).

S1.4 Simulation Analysis and Influence of Taper Angle of the Telescopic Cavity

The study of the telescopic cavity from the perspective of the taper angle can be divided into two aspects: adjusting the taper angle of the telescopic cavity while fixing the cross-sectional shape of the middle symmetry plane. Adjusting the taper angle of the telescopic cavity while fixing the shape of the air inlets/outlets at both ends.

Investigating the Effect of Different Taper Angles on Telescopic Cavity Performance (Fixed Annular Cross-Section of the Middle Symmetry Plane)

This section analyzes the influence of the taper angle on the normal tensile force at the right end of the telescopic cavity when its cross-section is fixed. Since the telescopic unit is axisymmetric, the left half is first defined. As shown in Fig. S4A, the entire side surface converges toward the center to form a cone, and a section reveals a conical shape. For a cone, the taper refers to the ratio of the diameter of the base circle to the height. For a truncated cone (frustum), the taper refers to the absolute difference between the diameters of the upper and lower bases divided by the height of the frustum. The taper angle is denoted as α, and the included angle β is the complement of half of α. Thus, changing β changes the magnitude of α. In this section, the annular cross-section of the middle symmetry plane is fixed (i.e., the length D in the figure is kept constant, so the size of the middle annulus remains unchanged). For better representation and understanding, β is used as the parameter. Meanwhile, due to structural constraints and matching with the size of the air inlets/outlets,

the tested values of β are 36°, 39°, 42°, 45°, 48°, and 51°, corresponding to α values of 108°, 102°, 96°, 90°, 84°, and 78°. Other parameters remain consistent.

As shown in Fig. S4B, in the simulation, all telescopic cavities with different β angles (under a fixed annular cross-section of the middle symmetry plane) converge normally. Their deformation process is like that in the previous section and will not be elaborated on here.

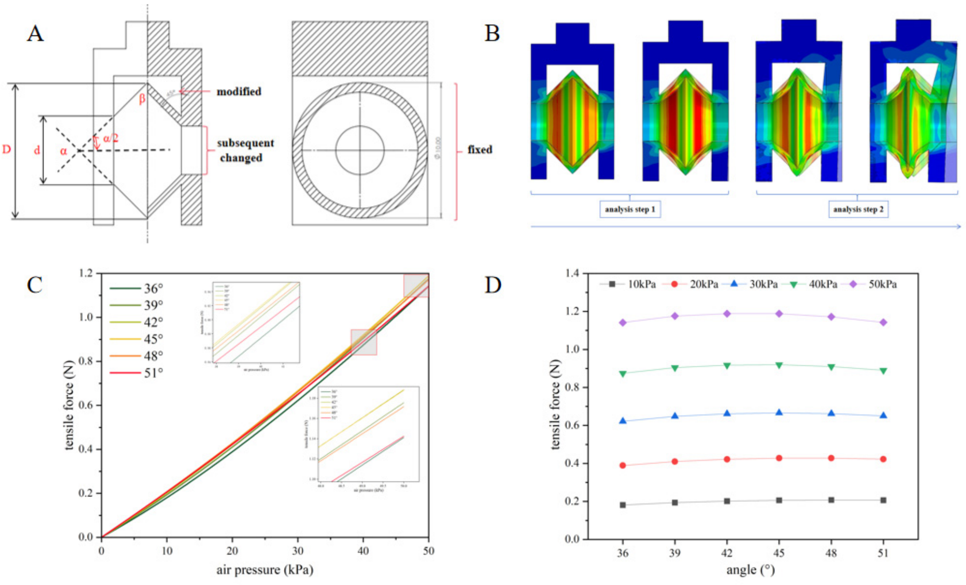

Fig. S4: A Telescopic cavity with β as the parameter (fixed middle annular cross-section);

B simulation deformation process of the telescopic cavity (fixed middle annular cross-section,

β = 45°) under -50 kPa; C normal tensile force performance of the telescopic cavity at the same β under different pressures; D normal tensile force performance of the telescopic cavity at the same pressure under different β.

As shown in Fig. 4C-D, when the annular shape of the middle symmetry plane is fixed, changing the magnitude of β has a minor impact on the normal tensile force of the telescopic cavity, with little overall change. At -50 kPa, the maximum difference in normal tensile force occurs between β=42°(1.1880 N) and β=36° (1.1412 N), with a difference of 0.0468 N. At -40 kPa, the maximum difference occurs between β=45° (0.9199 N) and β=36°(0.8743 N), with a difference of 0.0456 N. At -50 kPa, the difference between β=42°(1.1885 N) and β=45°(1.1880 N) is only 0.005 N. At -40 kPa, the difference between β=42°(0.9176 N) and β=45°(0.9199 N) is 0.0023 N (negligible). After fixing the annular shape of the symmetry plane, the structures on both sides of the symmetry plane can

be regarded as two frustums. Although the pressure acting on the telescopic cavity surface is the same, the surface area of the frustums on both sides (exposed to atmospheric pressure) does not change significantly. Thus, changing β (i.e., changing the taper angle α of the frustums) results in only minor differences in the generated normal tensile force.

Investigating the Effect of Different Taper Angles on Telescopic Cavity Performance (Fixed Shape of Air Inlets/Outlets at Both Ends)

It was found that changing β has a weak impact on the telescopic cavity when the middle symmetry plane is fixed. Here, the shape of the air inlets/outlets at both ends is fixed (i.e., the size d in the figure is kept constant), and the effect of changing β on the telescopic cavity performance is investigated (Fig. S5A). Owing to the structural constraints of the telescopic cavity height, the selected test parameters for the included angle β are 36°, 39°, 42°, 45°, 48°, and 51°, with the corresponding values of α being 108°, 102°, 96°, 90°, 84°, and 78°. All other settings remain consistent.

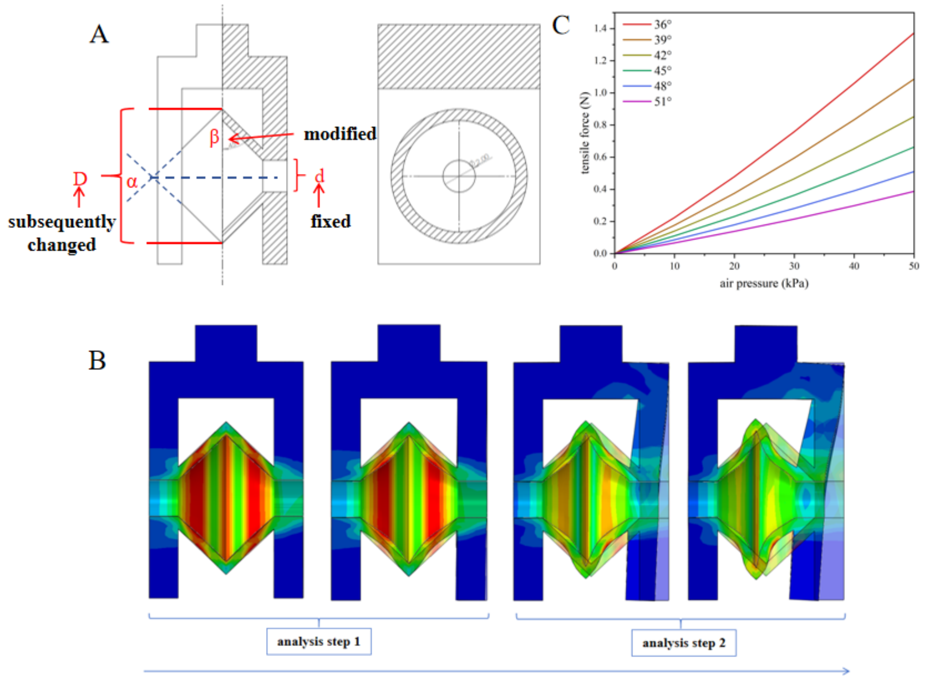

Fig. S5: A Identification of W/L ratio in the telescopic cavity (fixed air inlet/outlet shape at both ends); B simulation of the telescopic cavity (fixed air inlet/outlet shape at both ends, β=45°) under -50 kPa; C normal tensile force curves of telescopic cavities with different β angles (fixed air inlet/outlet shape at both ends) under different pressures.

In the simulation, all telescopic cavities with different β angles (under fixed air inlet/outlet shapes at both ends) converge normally (S5B). As shown in Fig. S5C, when the size/shape of the air inlets/outlets at both ends is kept constant, changing β has a significant impact on the normal tensile force of the telescopic cavity. At -50 kPa, the normal tensile forces at the right end are 1.37 N (β=36°) and 1.09 N (β=39°); the force for β=51°is only 0.39 N (a difference of 0.98 N from the maximum value). At -40 kPa, the normal tensile forces are 1.05 N (β=36°) and 0.83 N (β=39°); the force for β=51° is 0.30 N (a difference of 0.65 N from the maximum value). After fixing the size/shape of the air inlets/outlets at both ends, the structures on both sides of the symmetry plane are treated as two frustums. Changing β (i.e., changing the taper angle α of the frustums) leads to a significant change in the surface area of the frustums (exposed to atmospheric pressure). As β decreases from 51°to 36°, the surface area of the telescopic cavity in contact with the external environment gradually increases. Since the pressure on the cavity surface remains the same, the tensile force at the right end of the telescopic cavity (when the left end is fixed) gradually increases as β decreases.

S2 Structural Design and Friction Coefficient of the Suction Cup

The adsorption force of the suction cup mainly originates from the normal force generated by the internal–external pressure difference. Meanwhile, the skirt structure at the outer edge of the suction cup fits closely with the wall surface, forming a vacuum-sealed area inside the suction cup. After the air inside the suction cup is extracted, the external atmospheric pressure acts on the suction cup surface, generating a normal force that drives the fixed end of the entire suction cup to move toward the adherent wall surface—until the pressure on the suction cup surface and the external atmospheric pressure reach a new force balance.

As shown in Fig. S6, the coverage area of the suction cup projected onto the contact surface is defined as the theoretical contact area (area S). During the actual negative-pressure adsorption process, the region where the suction cup

contacts the surface is the actual contact area (area S1), and the non-contact region is the vacuum area (area S2). Let P_0 denote the external atmospheric pressure and P_v denote the air pressure in the vacuum area. Since the overall size of the flexible attachment device (and thus the suction cup's size and thickness) is small, the influence of its weight is neglected.

The actual forces acting on the suction cup include the following: the normal force at the fixed end, atmospheric pressure, contact stress at the boundary between the internal vacuum and the contact area (including shear stress at the boundary and normal stress during actual contact), air pressure in the inner cavity, and air pressure in the vacuum area. During force balance, the resultant force of the actual contact region (denoted N) under the normal force is defined as the contact pressure σ (as shown in the figure), which can be expressed as follows:

$$N = \oiint_{S1} \hat{y}\, \sigma dS \tag{S1}$$

The pressure F_{p0} exerted by the atmospheric pressure on the outer surface of the suction cup is expressed as follows:

$$F_{p0} = \oiint_{\psi} P_0\, \hat{n}_{dS} dS \tag{S2}$$

The pressure F_{pv} exerted by the air pressure in the vacuum area of the suction cup is expressed as follows:

$$F_{pv} = \oiint_{\phi} P_v\, \hat{n}_{dS} dS \tag{S3}$$

Here, Ψ represents the outer surface of the suction cup, ϕ represents the inner surface of the suction cup, $\hat{y}$ is the unit vector along the y-direction, and $\widehat{n_{dS}}$ is the unit normal vector on the infinitesimal area dS (pointing perpendicularly toward the vacuum suction cup). The difficulty in analyzing the adsorption force lies in modeling the elastic force of the suction cup. Due to the suction cup's shape, its normal deformation is more significant than tangential deformation. Thus, this study focuses on the force balance condition of the suction cup in the normal direction (i.e., along the y-axis).The pressure exerted by the air pressure on the suction cup (Eqs. S1 and S2) is projected along the y-axis as follows:

$$F_{p0}^{y} = F_{p0} \cdot \hat{y} = P_0(S_1 + S_2) \tag{S4}$$

$$F_{pv}^{y} = F_{pv} \cdot \hat{y} = P_v S_2 \tag{S5}$$

As shown in Fig. S6, a vertical section separates the vacuum area and the actual contact area. By isolating these two regions, the shear stress τ generated

by the elastic action of the skirt at the y-position of the actual contact section γ can be used to represent the vertical component of the internal elastic force:

$$f_e^y = \oiint_\gamma \tau \, dS \tag{S6}$$

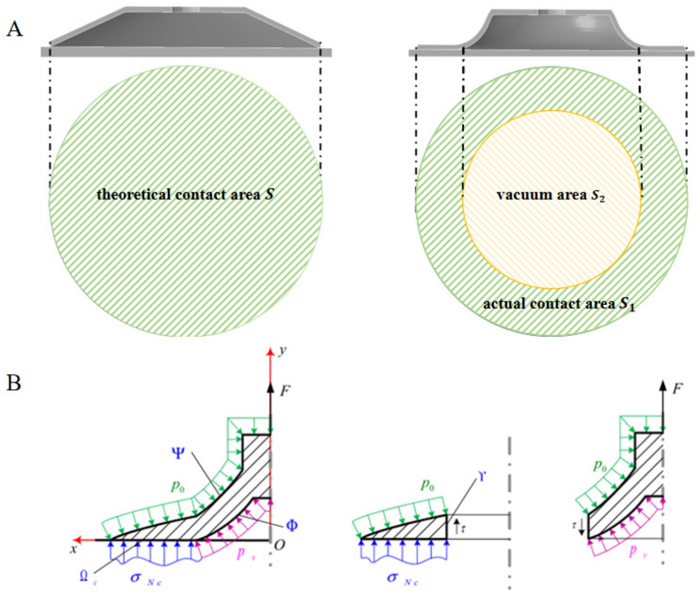

Fig. S6: Contact state and force analysis diagram of the suction cup and the adhered wall surface

(Note: a contact state of the suction cup on the adhered wall surface under normal pressure and negative pressure; B force analysis diagram of the suction cup under negative pressure).

Under negative pressure conditions, the bending deformation of the outer surface of the suction cup induces a large number of tensile variables along the generatrix direction of the suction cup, which renders the calculation extremely difficult. In addition, since the material of the suction cup is a hyperplastic body that can undergo large deformation under external forces and still recover its original shape, the force and energy required for its additional deformation are neglected in the following calculations. Therefore, from the perspective of the suction cup as a whole, the force balance can be expressed as follows:

$$P_0(S_1 + S_2) = F_n + P_v S_2 + N \tag{S7}$$

where F_n is the normal force generated by the suction cup under negative pressure when the palm center of the attachment device fixes the suction cup.

The force balance state of the suction cup in the actual contact area is as follows:

$$N = P_0 S_1 - f_e^y \tag{S8}$$

The force balance state of the suction cup in the vacuum area is as follows:

$$P_0 S_2 = F_n + P_v S_2 - f_e^y \tag{S9}$$

In summary, the normal force generated by the suction cup under negative pressure is related to the pressure difference between the inner and outer surfaces, the area of the vacuum region, and the internal elastic force of the suction cup, expressed as follows:

$$F_n = (P_0 - P_v) S_2 + f_e^y \tag{S10}$$

The tangential force of the suction cup refers to the contact force generated when a relative sliding tendency occurs between the suction cup and the contact surface. It is mainly composed of the friction force F_f, which is affected by the normal force between the suction cup and the contact surface ,as well as the friction coefficient, and is expressed as follows:

$$F_f = \mu \cdot F_n \tag{S11}$$

Suction cups made of different materials have different friction coefficients when in contact with smooth surfaces. In this study, the suction cup is made of Silicon 50A, and the smooth surface is an acrylic plate. To determine the friction coefficient μ_0 between them, a test specimen with a 10 mm × 10 mm contact surface was designed and fixed with a fixture, and preloads were set at 0.1 N intervals within the range of 0–1 N to measure the resulting tangential force (S7A). Linear fitting of the data yields a friction coefficient of approximately 0.838 between the Silicon 50A suction cup and the smooth acrylic plate (Fig. S7B).

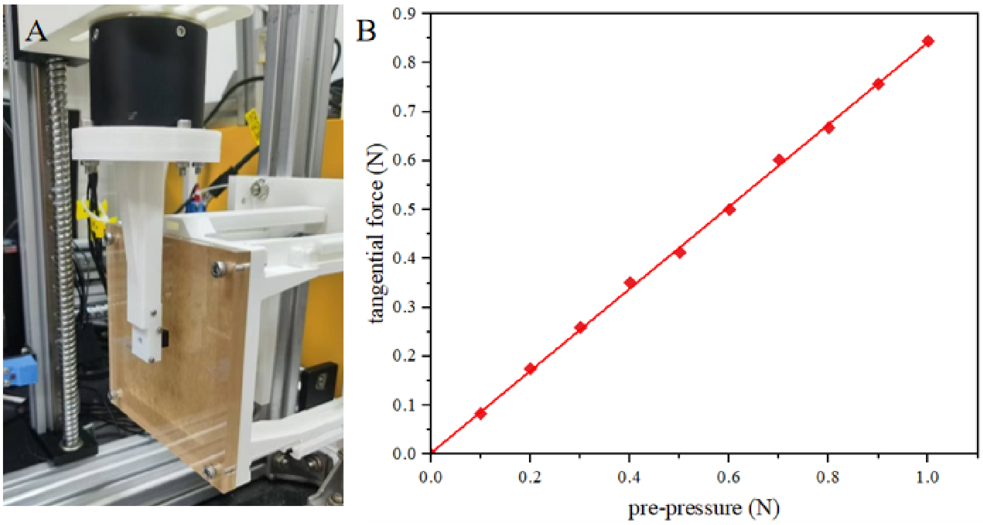

Fig. S7: Suction cup test bench and data diagram

(A: friction coefficient test bench for the suction cup and test plate; B: relationship between preload and tangential force of the suction cup).
